# Artificial Intelligence in the Imaging of Gastric Cancer: Current Applications and Future Direction

**DOI:** 10.3389/fonc.2021.631686

**Published:** 2021-07-21

**Authors:** Yun Qin, Yiqi Deng, Hanyu Jiang, Na Hu, Bin Song

**Affiliations:** ^1^ Department of Radiology, West China Hospital, Sichuan University, Chengdu, China; ^2^ Department of Laboratory Medicine, State Key Laboratory of Biotherapy, West China Hospital, Sichuan University, Chengdu, China

**Keywords:** gastric cancer, artificial intelligence, deep learning, hand-crafted radiomics, methodologies, clinical applications and challenges

## Abstract

Gastric cancer (GC) is one of the most common cancers and one of the leading causes of cancer-related death worldwide. Precise diagnosis and evaluation of GC, especially using noninvasive methods, are fundamental to optimal therapeutic decision-making. Despite the recent rapid advancements in technology, pretreatment diagnostic accuracy varies between modalities, and correlations between imaging and histological features are far from perfect. Artificial intelligence (AI) techniques, particularly hand-crafted radiomics and deep learning, have offered hope in addressing these issues. AI has been used widely in GC research, because of its ability to convert medical images into minable data and to detect invisible textures. In this article, we systematically reviewed the methodological processes (data acquisition, lesion segmentation, feature extraction, feature selection, and model construction) involved in AI. We also summarized the current clinical applications of AI in GC research, which include characterization, differential diagnosis, treatment response monitoring, and prognosis prediction. Challenges and opportunities in AI-based GC research are highlighted for consideration in future studies.

## Introduction

As one of the most common cancers, gastric cancer (GC) ranks as the top three in terms of mortality rate (1). The American Joint Commission on Cancer (8th Edition) for Gastric Cancer recommends computed tomography (CT) and endoscopic ultrasound for pretreatment TNM classification, whereas magnetic resonance imaging (MRI) and Positron Emission Tomography – Computed Tomography (PET-CT) are effective alternatives for metastasis evaluation. Despite the introduction of new techniques, the pretreatment diagnostic accuracy of GC varies from 40.8% to 98.1% (2–4). Efforts have also been made toward the prediction of histological type such as tumor differentiation grade and Lauren classification, based on enhancement pattern analysis, perfusion analysis, and spectral analysis, which have moderate discriminating performance and area under the curve (AUC) ranging from 0.697 to 0.891 (5–7). Given the importance of accurate pretreatment imaging evaluation and prognostic value of histopathological features, there is an urgent need for better diagnostic methods for treatment planning.

Fortunately, there has been considerable progress in artificial intelligence (AI) during the past decade, which offers promise for meeting these needs. Of all the AI techniques, hand-crafted radiomics and deep learning (DL) are the two most frequently applied methods for medical imaging and have shown the powerful capacity for converting mass medical images into minable data. With the ability to detect features that are invisible to human readers, hand-crafted radiomics and DL have demonstrated promising performance in tumor detection, characterization, and monitoring (8).

Therefore, we reviewed the published AI methodologies utilized in studies on GC imaging to provide an overview of the latest developments. This included data acquisition, lesion segmentation, feature design, and model construction. Furthermore, we summarize the representative clinical applications, knowledge gaps, and future directions. A total of 47 published AI studies on gastric cancer imaging were selected through MEDLINE (June, 2021), of which 45 were retrospective in design (36 single-center and 9 multicenter studies), while the remaining two were single-center prospective studies (**Table 1**). Imaging modalities varied across the studies. Specifically, 39 studies were performed using CT with only one studies based on dual-energy CT, six used MRI, and two used PET-CT (**Table 1**).

**Table 1:** Summary of published hand-crafted radiomics and deep learning studies on gastric cancer imaging.

No.	Authors	Year	Study objectives	Study design	No. of patients	Imaging Modality	Radiomics/Deep learning	Statistical analysis (feature selection and modelling)	Segmentation
1	Ba-Ssalamah et al. (9)	2013	Gastric tumors differentiation prediction	Retrospective, Single-center	48	CT	Radiomics	LDA+kNN	Manual
2	Sung Hyun Yoon et al. (10)	2016	HER2-positive and survival prediction	Retrospective, Single-center	26	CT	Radiomics	NA	Manual
3	Zelan Ma et al. (11)	2017	Gastric cancer and lymphoma differentiation	Retrospective, Single-center	70	CT	Radiomics	LASSO	Manual
4	Song Liu et al. (12)	2017	T and N staging prediction	Prospective, Single-center	80	MRI	Radiomics	ICC	Manual
5	Francesco Giganti et al. (13)	2017	Therapy response prediction	Retrospective, Single-center	34	CT	Radiomics	RF,LOOCV, Univariate analysis, Multivariate analysis	Manual
6	Shunli Liu et al. (14)	2017	Differentiation degree and Lauren classification prediction	Retrospective, Single-center	107	CT	Radiomics	ICC	Manual
7	Yujuan Zhang et al. (15)	2017	Histological differentiation prediction	Retrospective, Single-center	78	MRI	Radiomics	AOV, Spearman correlation analysis	Manual
8	Song Liu et al. (16)	2017	Nodal status prediction	Prospective, Single-center	87	MRI	Radiomics	Spearman correlation test, ICC	Manual
9	Song Liu et al. (17)	2017	Aggressiveness assessment	Retrospective, Single-center	64	MRI	Radiomics	Spearman correlation test	Manual
10	Francesco Giganti et al. (18)	2017	Association investigation between preoperative texture and OS	Retrospective, Single-center	56	CT	Radiomics	RSF ,Cox	Manual
11	Zhenhui Li et al. (19)	2018	Neoadjuvant chemotherapy response prediction	Retrospective, Single-center	47	CT	Radiomics	RF, NB, KNN, NNET, SVM, LDA, LASSO	Manual
12	Yuming Jiang et al. (20)	2018	Chemotherapy response and survival prediction	Retrospective, Multi-center	1591	CT	Radiomics	LASSO-Cox	Manual
13	Remy KlaassenI et al. (21)	2018	Treatment response prediction	Retrospective, Single-center	196	CT	Radiomics	RF, Pearson correlation	Manual
14	Zhen Hou et al. (22)	2018	Treatment response prediction	retrospective, Single-center	43	MRI	Radiomics	ICC, ACC, KNN, ANN	Manual
15	Yuming Jiang et al. (23)	2018	Survival and chemotherapy benefit prediction	Retrospective, Single-center	214	PET/CT	Radiomics	LASSO-Cox	Manual
16	Yuan Gao et al. (24)	2019	Metastatic lymph nodes prediction	Retrospective, Single-center	602	CT	Deep Learning	FR-CNN	Manual
17	Qiong Li et al. (25)	2019	Adverse histopathological status prediction	Retrospective, Single-center	554	CT	Radiomics	LASSO	Semiautomatic
18	Wujie Chen et al. (26)	2019	Metastatic lymph nodes prediction	Retrospective, Single-center	146	MRI	Radiomics	LASSO, LVQ	Manual
19	Yumin Jiang et al. (27)	2019	pN stage Prediction	Retrospective, Multi-center	1689	CT	Radiomics	LASSO	Manual
20	Qiu-Xia Feng et al. (28)	2019	Metastatic lymph nodes prediction	Retrospective, Single-center	490	CT	Radiomics	SVM	Manual
21	Yue Wang et al. (29)	2019	Tumor invasion prediction	Retrospective, Single-center	244	CT	Radiomics	ICC, RF	Semiautomatic
22	Wuchao Li et al. (30)	2019	OS prediction	Retrospective, Single-center	181	CT	Radiomics	ICC, LASSO-Cox	Manual
23	Xujie Gao et al. (31)	2020	Metastatic lymph nodes prediction	Retrospective, Single-center	463	CT	Radiomics	ICC, LASSO	Manual
24	Di Dong et al. (32)	2020	Prediction of the number of lymph nodes metastasis	Retrospective, Multi-center	679	CT	Radiomics, Deep learning	SVM, ANN, RF, DLRN	Manual
25	Xujie Gao et al. (33)	2020	Tumor-infiltrating Treg cells and outcome prediction	Retrospective, Single-center	165	CT	Radiomics	ICC, LASSO	Manual
26	Xiaofeng Chen et al. (34)	2020	Lymphovascular invasion and clinical outcome prediction	Retrospective, Single-Center	160	CT	Radiomics	ICC, SPM, LASSO	Manual
27	Na Wang et al. (35)	2020	HER2 over-expression status prediction	Retrospective, Single-Center	460	CT	Radiomics	ICC, Logistic	Manual
28	Xujie Gao et al. (36)	2020	Metastatic lymph nodes prediction	Retrospective, Single-center	768	CT	Radiomics	ICC, LASSO	Manual
29	Jing Li et al. (37)	2020	Lymph node metastasis risk prediction	Retrospective, Single-Center	204	CT	Radiomics, Deep Learning	ICC, ANN, KNN, RF, SVM	Manual
30	Yue Wang et al. (38)	2020	Lymph node metastasis prediction	Retrospective, Single-Center	247	CT	Radiomics	ICC, RF	Semiautomatic
31	Yue Wang et al. (39)	2020	Intestinal-type gastric adenocarcinomas distinction	Retrospective, Single-Center	187	CT	Radiomics	ICC, RF	Semiautomatic
32	Shunli Liu et al. (40)	2020	Occult peritoneal metastasis prediction	Retrospective, Single-center	233	CT	Radiomics	ICC, ACC, multivariate logistic regression	Manual
33	Aytul Hande Yardimci et al. (41)	2020	T and N stages and tumor grade prediction	Retrospective, Single-center	114	CT	Radiomics	ICC, LDA	Manual
34	Jing Yang et al. (42)	2020	Lymph node metastasis prediction	Retrospective, Single-center	170	CT	Radiomics	Pearson correlation analysis ,SFFS, logistic	Manual
35	Kai-YuSun et al. (43)	2020	Neoadjuvant chemotherapy response and survival prediction	Retrospective, Single-center	106	CT	Radiomics	SVM, PCA, Cox	Manual
36	Xiaofeng Chen et al. (34)	2020	Lymphovascular invasion and outcome prediction	Retrospective, Single-center	160	CT	Radiomics	ICC,SPM,LASSO	Manual
37	Wenjuan Zhang et al. (44)	2020	Early recurrence prediction	Retrospective, Multi-center	669	CT	Radiomics, Deep Learning	ICC, CV, DCNN	Manual
38	Yuming Jiang et al. (45)	2020	Tumor immune microenvironment and outcome prediction	Retrospective,Multi-center	1778	CT	Radiomics	Logistic	Manual
39	Liwen Zhang et al. (46)	2020	OS prediction	Retrospective, Multi-center	518	CT	Radiomics, Deep Learning	Cox	Manual
40	Xiao-Xiao Wang et al. (47)	2020	Lauren classification prediction	Retrospective, Single-center	539	CT	Radiomics	LASSO, logistic regression	Manual
41	Bao Feng et al. (48)	2021	Primary gastric lymphoma and Borrmann type IV gastric cancer differentiation	Retrospective, Multi-center	189	CT	Radiomics, Deep Learning	U-net based DL model, ICC, LASSO logistic regression	Automated
42	Yi-Wen Sun et al. (49)	2021	Gastric cancer and gastric lymphoma differentiation	Retrospective, Single-center	79	PET/CT	Radiomics	NA	Manual
43	Rui Wang et al. (50)	2021	Gastric neuroendocrine carcinomas and gastric adenocarcinomas differentiation	Retrospective, Single-center	63	CT	Radiomics	LASSO	Manual
44	Rui-Jia Sun et al. (51)	2021	Serosa invasion evaluation	Retrospective, Single-center	572	CT	Deep Learning	ICC, LASSO,DCNNs	Manual
45	Xiang Wang et al. (23)	2021	Prognosis prediction	Retrospective, Single-center	243	CT	Radiomics	multivariate COX regression analysis, LASSO	Manual
46	Yuming Jiang et al. (52)	2021	Occult peritoneal metastasis prediction	Retrospective, Multi-center	1225	CT	Deep Learning	PMetNet	Manual
47	Siwen Wang et al. (53)	2021	Disease-free survival prediction	Retrospective, Multi-center	353	CT	Radiomics	LASSO, multivariate Cox regression	Manual

LDA, Linear Discriminant Analysis; knn, k-Nearest Neighbors; NA, Not Available; LASSO, Least Absolute Shrinkage and Selection Operator; ICC, Intra-class Correlation Coefficient; RF, Random Forest; LOOCV, Leave One Out Cross Validation; AOV, Analysis Of Variance; NB, Naive Bayes; NNET, Neural Networks; SVM, Support Vector Machine; ACC, Absolute Correlation Coefficient; ANN, Artificial Neural Networks; FR-CNN, Faster Region-based Convolutional Neural Networks; LVQ, Learning Vector Quantization; DLRN, Deep Learning Radiomic Nomogram; SPM, Spearman correlation analysis; SFFS, Sequential Forward Floating Selection; PCA, Principal Component Analysis; RSF, Random Survival Forest; DCNN, Deep Convolutional Neural Networks; pmetnet, Peritoneal Metastasis Network; OS, Overall Survival.

## Methodologies of Ai Studies On Gastric Cancer

### Data Acquisition

Image preprocessing accounts for the substantial heterogeneity introduced by different imaging modalities, scanning protocols, machine types, and manufacturers. Image intensity normalization and resampling are two mathematical techniques that are used widely for this purpose. Specifically, image intensity normalization is performed to transform the original image into a standardized form to reduce data variability between cohorts and to generate appropriate inputs for quantitative radiomic feature calculation (20, 27). Resampling is used to adapt the input shape of the model by transforming the original image into the target size by upsampling or downsampling (32, 33, 36, 44).

In addition to imaging data, clinicopathological data also play an important role in AI-based modeling and can be used to improve model performance. These factors included patient age, gender, body mass index, cancer antigen 72-4 (CA72-4), CA199, CA242, carcinoembryonic antigen, alpha-fetoprotein, tumor location, tumor size, and TNM stages (9–13, 15, 17, 18, 20–22, 24–28, 30–36, 38–45, 54).

### Lesion Segmentation

Segmentation of region of interests (ROIs) in AI analysis can be performed using manual, automatic, or semiautomatic methods. Among the included AI-based GC studies, 42 (89%) studies utilized manual segmentation methods, four (9%) applied semiautomatic methods (25, 30, 38, 39), and only one study (2%) used automatic method (48).

Manual segmentation, which is usually carried out by radiologists, involves placing rectangular/circular boxes that delineate the two-/three-dimensional (2D/3D) boundary of the whole lesion. In Di Dong et al.’s study, 2D ROIs were placed to cover the largest tumor area for predicting lymph node metastasis in locally advanced GC (32). Yue Wang et al. segmented the entire tumor and built a 3D-based hand-crafted radiomics model to diagnose intestinal-type gastric adenocarcinomas (39). In addition, Wenjuan Zhang et al. constructed a DL model on 18 layers of residual convolutional neural network (CNN) with squared segmentation of CT images to predict overall survival (OS) in GC patients (44). It is important to note that because subjective judgments regarding tumor boundaries can vary substantially among radiologists, manual segmentations by multiple radiologists at multiple time points are required to minimize intra- and inter-rater variability. In addition, intra- and interclass correlation coefficients and coefficients of variation are often calculated to evaluate the robustness and reproducibility of the extracted features (12, 14, 17, 22, 30, 31, 34, 36, 39, 41).

In contrast to manual segmentation, semiautomatic segmentation usually comprises two steps. First, several labeling points are marked by radiologists. Thereafter, the entire ROIs are generated automatically by computing devices, based on the labeling points. Satisfactory gastric lesion segmentation performance has been achieved using this approach (25, 30, 38, 39). All the four studies using semiautomatic segmentation employed the same software package (Frontier, Syngo via, Siemens healthcare), which applies a dichotomic classification algorithm to semiautomatically segment lesions from perinormal areas.

### Feature Extraction

After lesion segmentation, quantitative handcrafted engineer features can be calculated to profile the intrinsic characteristics of the ROI. Handcrafted engineer features can be categorized as first-order statistics, shape-based, or texture-based features. First-order statistics are used to describe the distribution of pixel/voxel intensities in the ROIs, shape-based features show the geometric properties of the ROIs, and texture-based features are gray level matrices that represent textural patterns in an image region. Commonly used manual engineered features are presented in **Table 2**.

**Table 2:** Commonly used manual engineered features in gastric cancer.

No.	Shape- based 3D features (n=17)	Shape- based 2D features (n=10)	Histogram features (n=19)	Textural features (n=75)				
				Gray Level Co-occurrence Matrix (GLCM) Features (n=24)	Gray Level Run Length Matrix (GLRLM) Features (n=16)	Gray Level Size Zone Matrix (GLSZM) Features (n=16)	Neighbouring Gray Tone Difference Matrix (NGTDM) Features (n=5)	Gray Level Dependence Matrix (GLDM) Features (n=14)
1	Mesh Volume	Mesh Surface	Energy	Autocorrelation	Short Run Emphasis (SRE)	Small Area Emphasis (SAE)	coarseness	Small Dependence Emphasis (SDE)
2	Voxel Volume	Pixel Surface	Total Energy	Joint Average	Long Run Emphasis (LRE)	Large Area Emphasis (LAE)	contrast	Large Dependence Emphasis (LDE)
3	Surface Area	Perimeter	Entropy	Cluster Prominence	Gray Level Non-Uniformity (GLN)	Gray Level Non-Uniformity (GLN)	busyness	Gray Level Non-Uniformity (GLN)
4	Surface Area to Volume ratio	Perimeter to Surface ratio	Minimum	Cluster Shade	Gray Level Non-Uniformity Normalized (GLNN)	Gray Level Non-Uniformity Normalized (GLNN)	complexity	Dependence Non-Uniformity (DN)
5	Sphericity	Sphericity	10th percentile	Cluster Tendency	Run Length Non-Uniformity (RLN)	Size-Zone Non-Uniformity (SZN)	strength	Dependence Non-Uniformity Normalized (DNN)
6	Compactness 1	Spherical Disproportion	90th percentile	Contrast	Run Length Non-Uniformity Normalized (RLNN)	Size-Zone Non-Uniformity Normalized (SZNN)		Gray Level Variance (GLV)
7	Compactness 2	Maximum 2D diameter	Maximum	Correlation	Run Percentage (RP)	Zone Percentage (ZP)		Dependence Variance (DV)
8	Spherical Disproportion	Major Axis Length	Mean	Difference Average	Gray Level Variance (GLV)	Gray Level Variance (GLV)		Dependence Entropy (DE)
9	Maximum 3D diameter	Minor Axis Length	Median	Difference Entropy	Run Variance (RV)	Zone Variance (ZV)		Low Gray Level Emphasis (LGLE)
10	Maximum 2D diameter (Slice)	Elongation	Interquartile Range	Difference Variance	Run Entropy (RE)	Zone Entropy (ZE)		High Gray Level Emphasis (HGLE)
11	Maximum 2D diameter (Column)		Range	Joint Energy	Low Gray Level Run Emphasis (LGLRE)	Low Gray Level Zone Emphasis (LGLZE)		Small Dependence Low Gray Level Emphasis (SDLGLE)
12	Maximum 2D diameter (Row)		Mean Absolute Deviation (MAD)	Joint Entropy	High Gray Level Run Emphasis (HGLRE)	High Gray Level Zone Emphasis (HGLZE)		Small Dependence High Gray Level Emphasis (SDHGLE)
13	Major Axis Length		Robust Mean Absolute Deviation (rMAD)	Informational Measure of Correlation (IMC) 1	Short Run Low Gray Level Emphasis (SRLGLE)	Small Area Low Gray Level Emphasis (SALGLE)		Large Dependence Low Gray Level Emphasis (LDLGLE)
14	Minor Axis Length		Root Mean Squared (RMS)	Informational Measure of Correlation (IMC) 2	Short Run High Gray Level Emphasis (SRHGLE)	Small Area High Gray Level Emphasis (SAHGLE)		Large Dependence High Gray Level Emphasis (LDHGLE)
15	Least Axis Length		Standard Deviation	Inverse Difference Moment (IDM)	Long Run Low Gray Level Emphasis (LRLGLE)	Large Area Low Gray Level Emphasis (LALGLE)		
16	Elongation		Skewness	Maximal Correlation Coefficient (MCC)	Long Run High Gray Level Emphasis (LRHGLE)	Large Area High Gray Level Emphasis (LAHGLE)		
17	Flatness		Kurtosis	Inverse Difference Moment Normalized (IDMN)				
18			Variance	Inverse Difference (ID)				
19			Uniformity	Inverse Difference Normalized (IDN)				
20				Inverse Variance				
21				Maximum Probability				
22				Sum Average				
23				Sum Entropy				
24				Sum of Squares				

As opposed to handcrafted features, DL features are derived directly from the artificial neural networks, which encode medical images into a series of feature maps to extract features that represent high-dimensional information that cannot be detected by human readers. Using this method, Yuan Gao et al. achieved a mean average precision value and AUC of 0.7801 and 0.9541 in predicting perigastric lymph node metastasis, based on faster region-based CNN (24).

Handcrafted features describe the morphology, intensity, and textural patterns of ROIs, whereas deep learning network can automatically learn non-handcrafted feature representations from sample images.

Furthermore, studies combining handcrafted engineer and DL features have been carried out to maximize model efficiency. In Wenjuan Zhang et al.’s study, three handcrafted features, six DL features, and several clinical factors were combined to construct a nomogram, which demonstrated AUCs of 0.806-0.831 in predicting postoperative early recurrence in GC patients (44).

### Feature Selection

Most commonly used feature selection methods are categorized into the filter, wrapper, or embedded methods. Among these approaches, filter-based methods (e.g., correlation analysis, analysis of variance) are the simplest methods and select features according to a mutual information criterion (12, 14, 42, 55). Wrappers (e.g., recursive feature elimination, sequential feature selection algorithms, and genetic algorithms) extract useful features based on classifier performance. Filters and wrappers are frequently combined to improve feature selection ability. Using Pearson correlation analysis and the sequential forward floating selection algorithm, Jing Yang et al. obtained optimal tumor and nodal hand-crafted radiomics features to construct a model, which demonstrated good predictive performance for GC metastasis (42). Embedded methods perform variable selection during the model training process. The least absolute shrinkage and selection operator (LASSO) is a classical and widely applied embedded method (11, 19, 25, 27, 31, 33, 34, 36, 45). Unlike the aforementioned methods, LASSO regression adds a penalty against complexity, which can enable the construction of a simple, yet effective model with a small number of features.

### Model Construction

Regarding modeling strategy, logistic regression models (e.g., multivariate logistic regression, LASSO regression) have been widely used in AI-based GC studies. Random forest and support vector machines (SVM) are also effective alternatives for model construction (19, 28, 32, 36, 43). In a multicenter study, Di Dong et al. proposed an AI model that integrated DL, hand-crafted radiomics, and clinical factors. Their model used various modeling methods, including SVM, artificial neural networks, random forest, Spearman’s correlation analysis, logistic regression analysis, and linear regression analysis, and demonstrated good predictive performance for lymph node metastasis in locally advanced GC (32).

**Figure 1:**
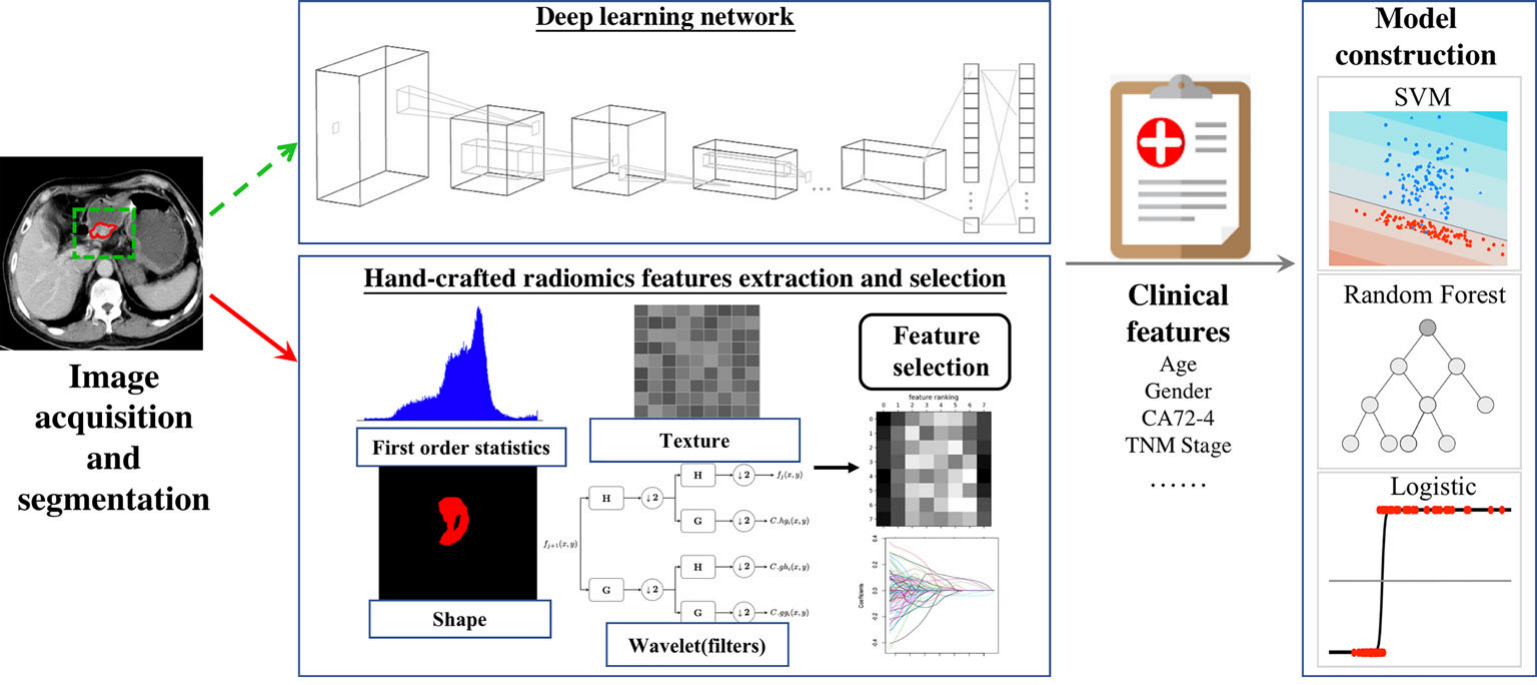
The workflow of hand-crafted radiomics and deep learning methodological process.

The above workflow and key methodologies of AI techniques in GC imaging are summarized in **Figure 1**.

## Clinical Applications of Hand-Crafted Radiomics and Deep Learning In Gastric Cancer

**Figure 2:**
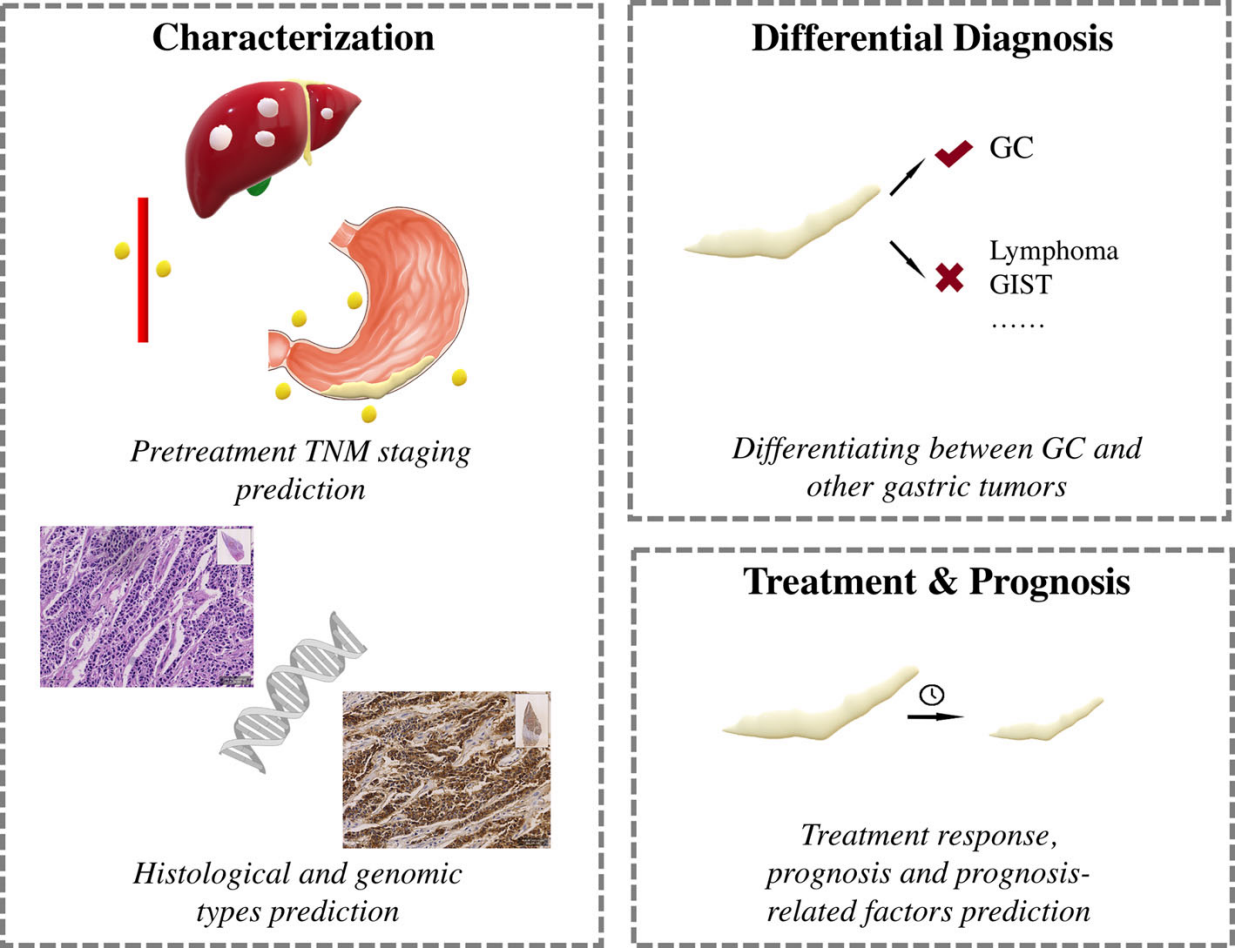
Clinical application of hand-crafted radiomics and deep learning in gastric cancer.

Major clinical applications of AI in GC research are shown in **Figure 2**.

### Characterization

The TNM classification is the most widely used staging system in GC, and pretreatment CT/MRI is vital for making optimal treatment decisions (56, 57). Considering its widespread application, most hand-crafted radiomics and DL studies have utilized CT images for preoperative prediction of TNM stages (24, 27, 28, 31, 32, 36–38, 40–42, 51, 52). Precise pretreatment TNM staging of lymph node metastasis is plagued by major obstacles because of discrepancies in traditional imaging features, such as shape, size, and enhancement patterns. Therefore, many researchers have been developing AI-based models to accurately predict lymph node status in GC patients (24, 27, 28, 31, 32, 36–38, 41, 42). While Yang et al.’s study combined tumor and nodal hand-crafted radiomics features (42), other studies selected only the tumor for the ROI (24, 27, 28, 31, 32, 36–38, 41). Of the 10 studies focusing on lymph node status, seven were designed to discriminate between N+ and N- (28, 31, 36–38, 41, 42), two to discriminate specific N stages (N0–3) (27, 32), and one with ambiguous lymph node status (24). Models based on hand-crafted radiomics, DL, or the combination of the two have shown AUC and C-indices of 0.79–0.95 in the training and 0.76–0.89 in the validation cohorts, respectively (24, 27, 28, 31, 32, 36–38, 41, 42). Three studies tested model efficacies for T stage prediction, where two aimed to discriminate T1/2 from T3/4 (29, 30, 41), and one to classify all T1–4 stages (25), with all yielding good discriminatory performance with AUCs ranging from 0.82 to 0.90. Liu et al. investigated venous CT images of primary tumors in advanced GC and built a hand-crafted radiomics model to predict occult peritoneal metastasis (40). Because of the popularity of CT, MRI has been used less frequently in GC patients, with only four studies focused on MRI-based prediction of TNM staging (12, 16, 17, 26). Using hand-crafted radiomics analysis, the authors found that diffusion-weighted imaging and apparent diffusion coefficient maps demonstrate potential in preoperative T and N staging for GC.

Using histopathological results as a reference, six studies explored the correlation between AI-based models and prognosis-related factors of tumor differentiation grade (9, 14, 15, 25), Lauren classification (14, 39, 47), and lymphovascular and neural invasion (14, 17, 25, 34). Two studies were based on MRI images (15, 17) and four were on CT images (14, 25, 34, 39), and all models exhibited good predictive ability for GC before operation. In addition, researchers carrying out immunohistochemistry studies have developed hand-crafted radiomics models to predict human epidermal receptor 2 status, which could serve as a noninvasive prediction tool for GC for selecting candidates suitable for Herceptin (10, 35). Furthermore, Gao’s hand-crafted radiomics model showed good performance in estimating tumor-infiltrating regulatory T (TITreg) cells, with AUCs of 0.85–0.88 in various cohorts (33).

### Differential Diagnosis

Five studies were conducted to differentiate between different gastric tumors (9, 11, 48–50). By applying texture analysis, Ba-Ssalamah et al. classified adenocarcinomas, lymphomas, and gastrointestinal stromal tumors from artery and portal venous CT images, respectively, and misclassification rates ranged from 0%–10% (9). Ma, Feng and Sun et al. focused specifically on differentiating Borrmann type IV GC from primary gastric lymphoma. By combining hand-crafted radiomics signatures, subjective CT findings, age, and gender, Ma’s model achieved a diagnostic accuracy of 87.1% (11). All these models demonstrated potential for accurate gastric tumor discrimination.

### Treatment Response and Prognosis

Neoadjuvant chemotherapy (NAC) can decrease tumor size and reduce mortality (58) and is recommended for potentially resectable advanced GCs. However, response rates of NAC vary among studies (59). In patients who do not benefit from NAC, the delay in surgery can lead to tumor progression and poor prognosis. Therefore, noninvasive selection of NAC responders before treatment is crucial for treating patients with advanced GCs. Three studies have utilized CT-based hand-crafted radiomics analysis build models to predict non-responders, which have yielded AUCs of 0.65–0.82 (13, 19, 43). Notably, Sun et al. demonstrated that their hand-crafted radiomics model performed better for NAC response prediction compared with a clinical model (43).

Chemotherapy and radiation therapy are two mainstays for advanced GCs. Three studies have been carried out to predict chemotherapy response (20, 21, 23). Jiang et al.’s model showed that higher scores of their CT-based hand-crafted radiomics signature indicated a favorable response to chemotherapy for stage II-III patients (20). Similarly, Jiang et al. built a Rad-score system based on hand-crafted radiomics features from PET images, where higher scores indicated chemotherapy responders (23). Klaassen et al. focused on individual liver metastases in esophagogastric cancers and developed a CT-based hand-crafted radiomics model to predict responsive lesions; the resulting AUCs ranged between 0.65–0.87 in various cohorts (21). Only one study tested model efficacy for radiotherapy responders in GC patients with abdominal cavity metastasis. Based on pretreatment CT images, Hou et al. constructed two prediction models with high accuracies ranging from 0.71 to 0.82 (22).

Prognosis is the research highlight of AI-based studies and numerous researchers have explored the potential of hand-crafted radiomics and DL features for prognosis prediction (10, 18, 20, 23, 25, 30, 33, 34, 43–46, 53, 60). Nine studies directly correlated hand-crafted radiomics and DL features with prognosis (10, 18, 20, 23, 30, 44, 46, 53, 60), and five constructed AI-based models to predict certain clinicopathological features, which were shown to be related to prognosis (25, 33, 34, 43, 45). Only one study extracted hand-crafted radiomics features from PET images (23), whereas all others used CT images (10, 13, 18–22, 25, 30, 33, 34, 43–46, 53, 60). Earlier studies reported OS-related hand-crafted radiomics features (10, 18), with later studies building hand-crafted radiomics and DL models that integrated hand-crafted radiomics features with and without clinicopathological features; these achieved good performance in OS, disease-free survival (DFS), and early recurrence prediction (20, 23, 30, 33, 44). Furthermore, hand-crafted radiomics models to predict clinicopathological features, such as TITreg cells, lymphovascular invasion, adverse histopathological status, tumor immune microenvironment, and NAC response have also been developed, which have yielded AUCs of 0.75–0.89 for predicting OS, DFS, and progression-free survival (25, 33, 34, 43, 45).

## Future Challenges and Opportunities

To date, numerous studies have demonstrated the prediction potential of hand-crafted radiomics and DL in GC characterization, differential diagnosis, treatment response, and prognosis. Despite the frequent application of MRI in clinical practice, it is not routinely recommended for GC evaluation. Most studies have focused on CT images and few have used MRI images. Considering its excellent resolution of soft tissue, MRI images may reveal more intrinsic tumor features and improve prediction. Therefore, future investigations should aim to include more patients undergoing MRI examinations for GC evaluation. Lymph node metastasis status is a key component of pretreatment and postoperative evaluation, and many studies have developed methods for pretreatment AI-based prediction, which include prediction of the existence of lymph node metastasis and N stage. However, there have not been any studies that have focused on individual lymph nodes, which is fundamental for precise pretreatment N stage evaluation and treatment plan modification during follow-up. We encourage future studies to focus on individual lymph node metastasis status prediction based on rigid pathological correlations. Moreover, few studies have analyzed the relationship between imaging features and treatment response. There is still a considerable knowledge gap in this field; further research is needed to improve patient selection and develop better treatment plans.

In addition, future efforts should continue to be actively pursued regarding the methodologies of AI. More intensive and standardized quality controls throughout the entire workflow of AI are warranted to meet this requirement. By analyzing a total of 77 hand-crafted radiomics-based oncology researches, Park et al. reported insufficient overall scientific quality of current hand-crafted radiomics studies (61). Similar dilemmas arose at every stage of GC from data acquisition, segmentation, feature extraction, feature selection, model construction to model performance reporting. In this context, compliance with widely-accepted quality systems [e.g. Hand-crafted radiomics Quality Score (RQS) (62), Transparent Reporting of a multivariable prediction model for Individual Prognosis Or Diagnosis (TRIPOD) (63), etc.] may offer appeal. In addition, prospective multi-institutional collaborations to establish well-curated databases and networks are encouraged in future studies. Furthermore, considering the inherent capacity of AI in analyzing parallel streams of information, including clinical and genomics characteristics (64–66), multi-omics studies which integrate these data may pave the way for better personalized and precision medicine. Collectively, we hope the fruit of these efforts could help to shift the landscape of AI in GC from exploratory research settings to routine clinical settings.

## Conclusion

GC has a high incidence and mortality rate, which have been the clinical research emphasis over the past decades. Hand-crafted radiomics and DL are emerging quantitative subsets of AI that have been widely utilized in medicine. The exploration of GC using hand-crafted radiomics and DL has led to promising results for every step of the clinical pathway. However, most studies have been retrospective, conducted in a single center, and analyzed using a single image modality, which have limited the utility of the constructed AI models. Therefore, further prospective and multicenter studies are needed to validate the models. Moreover, other imaging modalities, such as endoscopic ultrasound may be integrated into the models to further improve model efficacy.

## Author Contributions

BS and NH designed and supervised this study. YQ, YD, and HJ conducted the literature search, article selection, data extraction, data analyses and data interpretation. YQ and YD contributed to the conception of the study and drafted the manuscript. All authors contributed to writing of the manuscript and approved the final manuscript. YQ and YD contributed equally to this work. All authors contributed to the article and approved the submitted version.

## Funding

This work was supported by grants from the National Natural Science Foundation of China (82002569, 81902437); 1.3.5 Project for Disciplines of Excellence, West China Hospital, Sichuan University (ZYJC21006, ZYYC20003).

## Conflict of Interest

The authors declare that the research was conducted in the absence of any commercial or financial relationships that could be construed as a potential conflict of interest.
