# Supplementary material for: Artificial Intelligence in the Imaging of Gastric Cancer: Current Applications and Future Direction
Source: Front Oncol. 2021 Jul 21;11:631686. doi: 10.3389/fonc.2021.631686 (PMC8335156; doi:10.3389/fonc.2021.631686)
Supplement: Supplementary file 1 [file DataSheet_1.docx]

**Step1. Image acquisition and segmentation.**

After acquiring the sample images, preprocessing (e.g. image intensity normalization and resampling) is used to obtain compliant images. Thereafter, segmentation of region of interests (ROIs) can be performed in manual, automatic, or semiautomatic manners.

**Step2. Feature extraction and selection.**

Hand-crafted radiomics features or deep learning (DL) features can be calculated to profile the intrinsic characteristics of the ROIs. In specific, hand-crafted features are categorized as first-order statistics, shape-based, and texture-based features, with the filter, wrapper, or embedded methods used for feature selection. DL features are derived directly from the artificial neural networks.

**Step3. Model construction.**

Support vector machine, random forest and logistic regression analysis are commonly applied modelling algorithms. Clinical features could be further integrated to refine the AI models.
