# Supplementary material for: Artificial Intelligence in the Imaging of Gastric Cancer: Current Applications and Future Direction
Source: Front Oncol. 2021 Jul 21;11:631686. doi: 10.3389/fonc.2021.631686 (PMC8335156; doi:10.3389/fonc.2021.631686)
Supplement: Supplementary file 2 [file DataSheet_2.docx]

**Searching Strategy of MEDLINE:**

("radiomic"[All Fields] OR "radiomics"[All Fields] OR (("textural"[All Fields] OR "texturally"[All Fields] OR "texture"[All Fields] OR "texture s"[All Fields] OR "textured"[All Fields] OR "textures"[All Fields] OR "texturing"[All Fields] OR "texturization"[All Fields] OR "texturize"[All Fields] OR "texturized"[All Fields] OR "texturizing"[All Fields]) AND ("analysis"[MeSH Subheading] OR "analysis"[All Fields])) OR ("deep learning"[MeSH Terms] OR ("deep"[All Fields] AND "learning"[All Fields]) OR "deep learning"[All Fields]) OR ("artificial intelligence"[MeSH Terms] OR ("artificial"[All Fields] AND "intelligence"[All Fields]) OR "artificial intelligence"[All Fields])) AND "stomach neoplasms"[MeSH Terms]


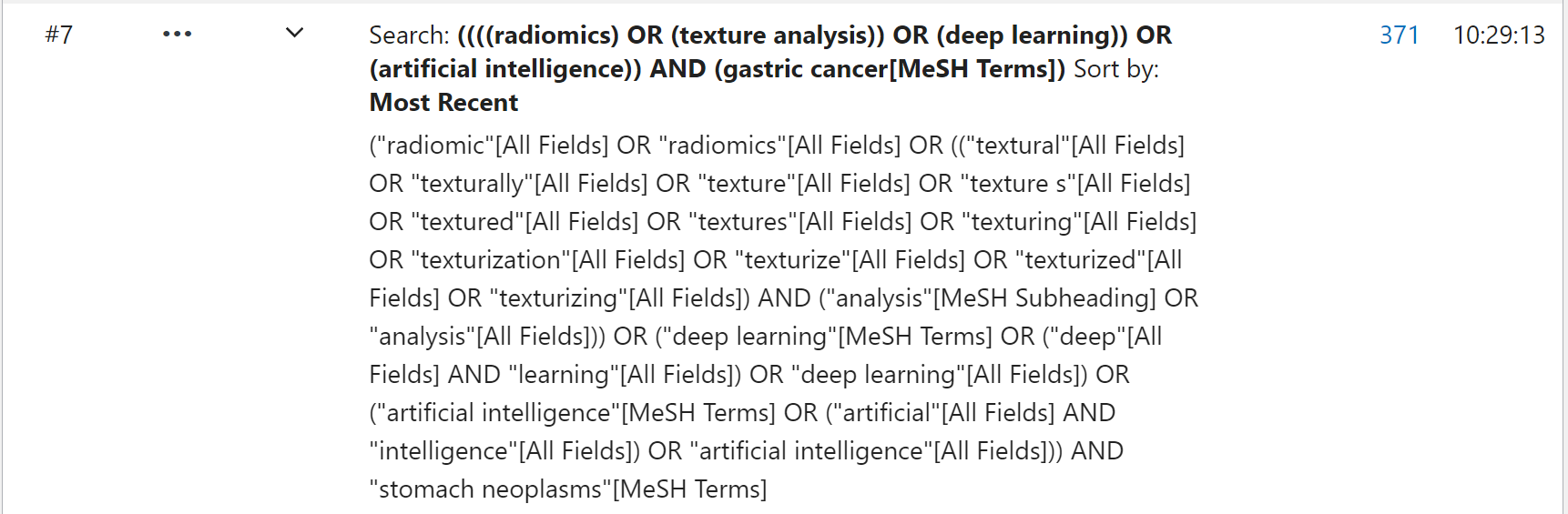


**Article Selection Process:**

**
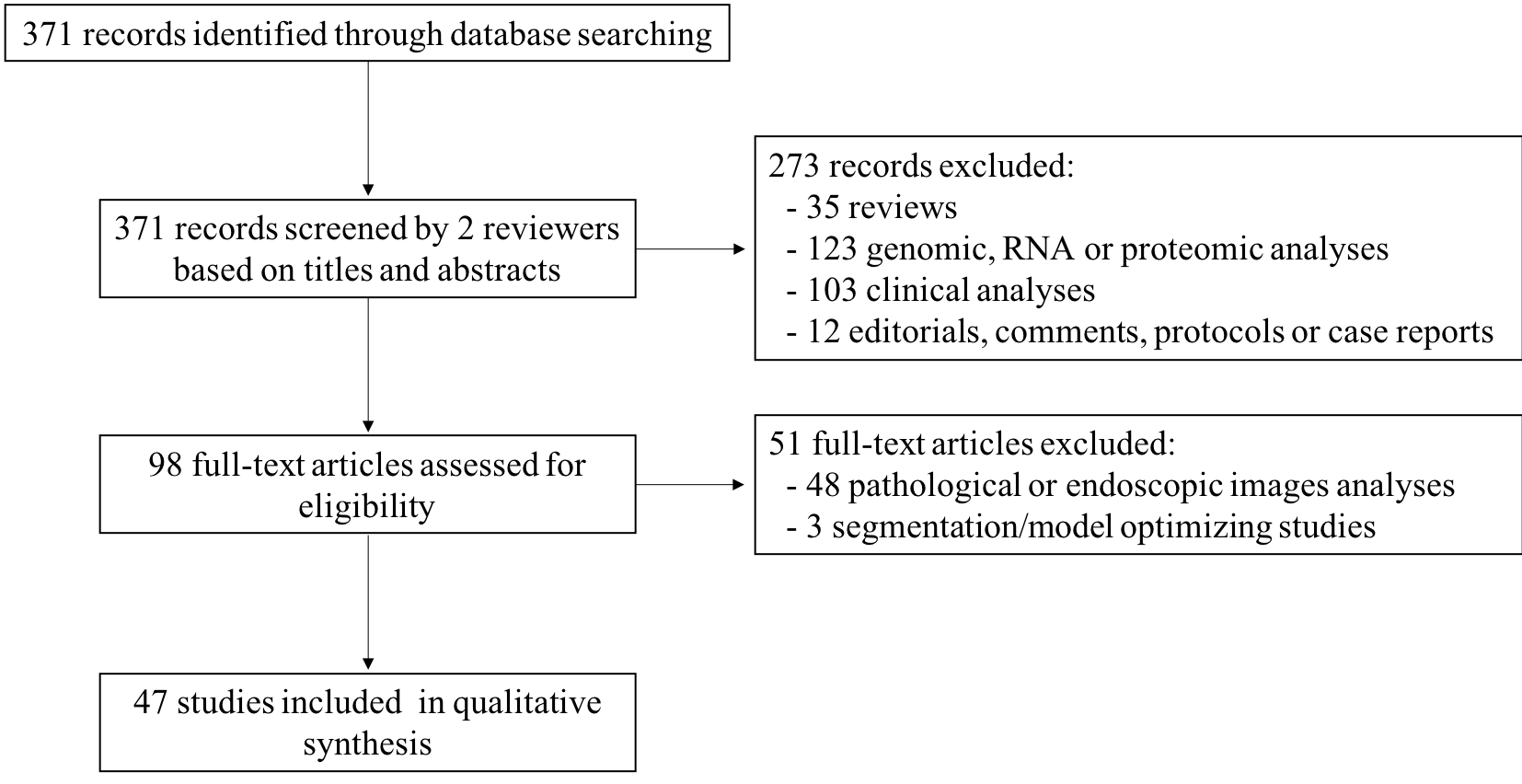
**
